# Population pharmacokinetics of FCN-159, a MEK1/2 inhibitor, in adult patients with advanced melanoma and neurofibromatosis type 1 (NF1) and model informed dosing recommendations for NF1 pediatrics

**DOI:** 10.3389/fphar.2023.1101991

**Published:** 2023-01-23

**Authors:** Yan Tan, Ailing Cui, Lixuan Qian, Chao Li, Zhuli Wu, Yuchen Yang, Pu Han, Xin Huang, Lei Diao

**Affiliations:** ^1^ Beijing Fosun Pharmaceutical Research and Development Co., Ltd., Shanghai, China; ^2^ dMed Biopharmaceutical Co., Ltd., Shanghai, China; ^3^ Fosun Pharma USA Inc., Princeton, MA, United States

**Keywords:** population pharmacokinetics, FCN-159, pediatric starting dose selection, rat sarcoma virus, mitogen-activated protein kinase pathway, neurofibromatosis type 1

## Abstract

**Objective:** FCN-159 is a highly active mitogen-activated extracellular signal-regulated kinase 1/2 (MEK1/2) inhibitor in patients with advanced melanoma and neurofibromatosis type 1 (NF1). We report a population pharmacokinetic (PopPK) model-based analysis of FCN-159 and its application to inform dose selection for NF1 pediatric trials.

**Methods:** PK data collected from patients with advanced melanoma and NF1 in two clinical studies (NCT03932253 and NCT04954001) were analyzed using a non-linear mixed effects model. The adult model was adapted by incorporating allometric scaling for PK projection in 2–17 years old children. Pediatric exposure in different body surface area (BSA) bins was simulated to identify nominal doses (i.e., dose amounts given as integers) and BSA bin cutoffs to achieve exposure comparable to adults’ optimal exposure across the entire pediatric BSA range.

**Results:** The final dataset consisted of 45 subjects with a total of 1030 PK samples. The PK of FCN-159 was well-described by a 2-compartment model with first-order linear elimination and delayed first-order absorption. Covariates, including BSA, age, sex, albumin, total protein, and cancer type, were identified as statistically significant predictors of FCN-159 disposition. Simulations based on the final model projected daily doses of 4 mg/m^2^ QD with optimized BSA bin cutoffs would allow fixed nominal doses within each bin and result in steady state exposure approximating the adult exposure observed at the recommended phase 2 dose (RP2D) in NF1, which is 8 mg QD.

**Conclusion:** The developed population PK model adequately described the PK profile of FCN-159, which was adapted using allometric scaling to inform dose selection for NF1 pediatric trials.

## 1 Introduction

Rat sarcoma virus (RAS) oncogene mutations and abnormal activation play an essential role in the development of melanoma and neurofibromatosis type 1 (NF1) (Nissan et al., 2014; Kiuru and Busam, 2017; Muñoz-Couselo et al., 2017). Activated RAS continuously activates a series of downstream signaling pathways, including mitogen-activated protein kinase (MAPK) pathway (RAS/RAF/MEK/ERK) (McCubrey et al., 2007; Mendoza et al., 2011), which is a promising therapeutic target in cancer (Braicu et al., 2019).

MEK inhibitors selectively inhibit mitogen-activated extracellular signal-regulated kinase 1/2 (MEK1/2) activity in individuals with v-raf murine sarcoma viral oncogene homolog B1 (BRAF) or RAS-positive malignancies or NF1, and impair signal transduction pathways that regulate proliferation and survival in cancer cells (Kuske et al., 2018; Klesse et al., 2020). Several clinical trials have demonstrated the benefits of MEK inhibitors (e.g., trametinib (Robert et al., 2019), cobimetinib (Gutzmer et al., 2020), mirdametinib (Weiss et al., 2021) and selumetinib (Gross et al., 2020) in melanoma and NF1. The development of MEK inhibitors, including combination strategies with immune checkpoint inhibitors, is crucial for patients with RAS-positive malignancies or NF1 (Tamura, 2021; Dixon-Douglas et al., 2022).

FCN-159 is a highly active MEK1/2 inhibitor that inhibits MEK phosphorylation with low inhibitory potential for other kinases (Cheng and Tian, 2017). The molecular weight of FCN-159 is 665.5. Preclinical studies revealed the inhibitory effect of FCN-159 on the proliferation of RAS/RAF mutant tumor cells, with antitumor efficacy in various murine xenograft tumor models (Lin et al., 2020). The first-in-human (FIH) study of FCN-159 in melanoma (FCN-159-001) (Si et al., 2019) and a phase I/II trial in NF1 (FCN-159-002) (Hu et al., 2022) demonstrated promising anti-tumor effects and tolerability in adult patients with NRAS mutant solid tumors. In melanoma FIH, thirty-three patients were enrolled across nine FCN-159 dose groups (0.2–15 mg). One DLT grade 3 folliculitis occurred in the 15-mg group and the maximum tolerated dose (MTD) as well as recommended phase 2 dose was determined as 12 mg (Si et al., 2019; Mao et al., 2022). In phase I study in NF1 patients, 19 patients were enrolled in 4 dose cohorts (4–12 mg). Four patients experienced DLTs of grade 3 folliculitis, which were reported in 1 (16.7%) patient receiving the 8-mg dose and 3 (100%) patients receiving the 12-mg dose. The MTD and RP2D were determined to be 8 mg (Hu et al., 2022).

FCN-159 tablet was readily absorbed following oral administration in adults, with time to maximum concentration within 3 h. FCN-159 exhibits approximately dose proportional increases in exposure over the dose range of 0.2–15 mg and is metabolized *via* multiple pathways, primary by cytochrome P450 (CYP) 3A4 and to a lesser extent by CYP2C19 and CYP2C8 (Mao et al., 2022).

Pediatric patients represent a large proportion of NF1 cases (McGaughran et al., 1999; Kallionpää et al., 2018). So far, only selumetinib was approved for treating pediatric patients with NF1. A lower daily dose of FCN-159 without food intake restriction could potentially provide a better safety profile and improve patient compliance in pediatrics. Since the recommended doses of FCN-159 in adults have been established previously (Hu et al., 2022), this study aimed to predict dose(s) in pediatrics using the model informed approach, to minimize the risk of over- or underdosing in the first pediatric trial. A population PK model was developed using adult data and adapted to establish a pediatric model by incorporating allometric scaling. By using adults’ optimal exposure as a reference, the pediatric model was used to simulate FCN-159 exposure in pediatric NF1 patients under different BSA based dosing regimens aiming to achieve comparable exposure and guide dose selection in pediatrics.

## 2 Materials and methods

### 2.1 Ethics approval and patient consent

All study protocols were approved by the institutional review board of each study center and conducted in accordance with Good Clinical Practice (GCP) and the guiding principles of the Declaration of Helsinki. All study participants provided signed informed consent before enrollment.

### 2.2 Data collection

FCN-159 plasma concentrations from 2 phase I clinical trials, including FCN-159-001 (NCT03932253) and FCN-159-002 (NCT04954001), were collected and pooled in the PopPK analysis (Supplementary Table S1). FCN-159-001 was a multi-center, open-label, single-arm phase Ia/Ib study evaluating the safety, tolerability, pharmacokinetics, and preliminary anti-tumor activity of FCN-159 in advanced melanoma patients with aberrant NRAS (phase Ia) and NRAS mutation (phase Ib). In the dose escalation portion, the dosing regimens were 0.2, 0.5, 1, 2, and 4 mg QD for 21 days, and 6, 8, 12, and 15 mg QD for 28 days in every 28-day cycles. FCN-159-002 was a multi-center, open-label, single-arm phase I/II study evaluating the safety, tolerability, pharmacokinetics, and anti-tumor activity of FCN-159 in adult and pediatric patients with NF1. The dosing regimens were 4, 6, 8, and 12 mg QD for 28 days in 28-day cycles (Mao et al., 2022). Both studies were conducted in China with Chinese patients and FCN-159 tablets were taken under fasting state.

In these studies, FCN-159 plasma concentrations were analyzed by a validated liquid chromatography-tandem mass spectrometry (LC-MS-MS) method with an assay range of 0.2–200 ng/ml.

### 2.3 Software

Analysis data assembly, descriptive statistics, and plots were programmed using R version 4.0.3 (http://www.r-project.org/). Population PK analyses and all related simulations were performed with NONMEM version 7.4 (ICON Development Solutions, Ellicott City, Maryland) and Perl-speaks-NONMEM version 5.0.0 (PsN, Uppsala University, Uppsala, Sweden). NONMEM was compiled based on GNU Fortran (GCC) 4.6.3 and was called by Strawberry Perl (64-bit) 5.28.1.1 and Perl-speaks-NONMEM 4.8.1.

### 2.4 PopPK modeling

#### 2.4.1 Base model

Following graphical assessment, models with one, two and three compartments were utilized to describe the concentration-time profiles of FCN-159. Several absorption models were tested, including first-order, sequential zero- and first-order, and transit compartment models, with or without a lag absorption time. The compartmental models were parameterized using apparent clearance(s) and volume(s) of distribution. The first-order conditional estimation method with interaction (FOCE-I) was used for parameter estimations. The base PopPK model was selected among candidate models by comparing the Akaike information criterion (AIC), goodness-of-fit, plausibility of parameter estimates, precision of parameter estimates, minimum objective function value (OFV), and eta shrinkage values. The two-compartment model with first-order elimination and first-order absorption with lag time was selected as the structural model. The residual variability was modeled as combined additive and proportional errors. The inter-individual variability (IIV) model was described as a lognormal distribution.

#### 2.4.2 Covariate model

Pre-specified covariates were rationalized by their physiological relevance, and their effects on PK parameters were evaluated. BSA or body weight (BW), sex, age, and renal/hepatic function markers, including creatinine clearance (CrCL), aspartate aminotransferase (AST), alanine aminotransferase (ALT), bilirubin, albumin, and total plasma protein, were tested for their effects on FCN-159 clearance. Cancer type was tested in relation to absorption parameters. BSA or BW, sex, age, red blood cell count, albumin, and total plasma protein were tested for their effects on volume of distribution.

To extrapolate the adult model for pediatric study simulation, allometric scaling based on body size was implemented. BW and BSA were tested as default covariates based on apparent clearance type (systemic clearance, CL; intercompartment clearance, Q) and volume of distributions (central compartment, V_C_; peripheral compartment, V_P_) using power functions. BW was normalized to 60 kg, and BSA to the median BSA value of 1.66 m^2^.

$$P_{i}=\theta_{TV}\cdot {\left(\frac{{Body\;size}_{i}}{Median\left({Body\;size}_{i}\right)}\right)}^{\theta }\exp\left(\eta_{i}\right)$$

where $P_{\;i}$ is the individual predicted PK parameter (i.e., CL, Q, V_C_, or V_P_); $\theta_{TV}$ is the typical value of the PK parameter; θ represents body size based exponent; $\eta_{i}$ is an independent and normally distributed between-subject random variable with a mean of 0 and a variance of $\omega_{\theta }^{2}$ (i.e., $\eta ∼N\left(0,\omega_{\theta }^{2}\right)$ ). ${Body\;size}_{i}$ is a body size measurement (i.e., body weight or BSA). $Median\left({Body\;size}_{i}\right)$ is the median body size. The exponent term *θ* either used empirical values (i.e., BW raised to the power of 0.75 for clearance and 1.0 for volume) or were fitted for the adult data. The best allometric model was selected based on OFV, the plausibility of parameter estimates, and the precision of parameter estimates.

Continuous covariates were explored using a linear model centered at the median:

$$P_{j}=\theta_{0}+\theta_{1}\cdot \left(X_{1}-Median\left(X_{1}\right)\right)$$

Other functional forms, including log-linear and exponential models, were evaluated if suggested by the covariate-PK parameter relationship:

$$P_{j}=\theta_{0}\cdot {\left(\frac{X_{1}}{Median\left(X_{1}\right)}\right)}^{\theta_{1}}$$

or

$$P_{j}=\theta_{0}\cdot \exp\left(\theta_{1}\left(X_{1}-Median\left(X_{1}\right)\right)\right)$$

$P_{j}$ is the jth parameter, i.e., $\theta_{1}$ is the slope associated with the covariate; $X_{1}$ is a covariate, and $Median\left(X_{1}\right)$ is the median value of the covariate.

Binary and multi-categorical covariates with the proportion of every class ≥10% of the total population were explored using a categorical model.

$$P_{j}=\theta_{0}\cdot \left(1+\theta_{1}\cdot X_{1}\right)$$

For a binary covariate, *θ_1_* is the effect of the categorical covariate on the PK parameter with respect to the reference category.

For multi-categorical covariates, a class accounting for <10% of the total population would be combined with similar items; otherwise, it would not be investigated.

BSA as the allometric term was included in the model by default without statistical test as mentioned above. All other candidate covariates were screened by a univariate stepwise approach. In the first step, covariates that were potential predictors in the model were identified one by one. The covariate with the most significant reduction in the OFV along with chi-square test *p* < 0.05 was included into the model. The inclusion steps were repeated until no additional covariates could meet the *p* < 0.05 criterion in the chi-square test. In the backward elimination steps, if removing a covariate had the least reduction in the OFV among all covariates and did not significantly worsen the OFV based on chi-square test *p* < 0.01, the covariate was then excluded from the model. The exclusion steps were repeated until no additional covariates could be removed from the model. After the covariate model was finalized, the IIV model was reassessed (e.g., dropping poorly estimated or close to zero IIVs from the model). A non-diagonal variance-covariance matrix for IIVs was also evaluated. Forest plots were constructed by varying values for one covariate at a time to determine the effects of individual covariates on PK parameters and FCN-159 exposure. For forest plots, categorical covariates were assessed at each level, while continuous covariates were assessed at their reference values (i.e., median values) and the corresponding 5th, 25th, 75th, and 95th percentiles.

### 2.5 Model evaluation

The stability of the model was verified by condition number (the ratio of the maximum eigenvalue to the minimum eigenvalue of the variance-covariance matrix). The model was considered stable with an eigenvalue of less than 1000. The robustness of the model and parameter estimates were assessed by the bootstrap method. Among 1000 bootstrap runs, a success rate greater than 80% was regarded as acceptable. Parameter estimates close to the median of bootstrap estimates suggests stable parameter estimates. The model’s predictability was verified by visual predictive check (VPC) (Yano et al., 2001; Bergstrand et al., 2011). In the VPCs, based on 1000 simulated replicates, prediction intervals for individual predicted concentration-time profile percentiles (median, 5th, and 95th percentiles) were superimposed on those percentiles (median, 5th, and 95th percentiles) derived from the observed data to evaluate if the simulated model could replicate the observed dataset.

### 2.6 Simulation

The developed adult population PK model was adapted using allometric scaling to inform pediatric dose selection in children aged 2–17 years. Simulations were performed to identify pediatric doses that would result in exposure comparable to the observed adult exposure at selected therapeutic dose of 8 mg. Virtual pediatric patients were simulated using a uniform BSA distribution between 0.55 and 2.15 m^2^. The stochastic simulation included random resampling from IIVs and residual error distributions. IIVs and residual error distributions were assumed to be the same between adults and pediatric patients. The simulation was also assumed similar bioavailability between adults and children. The reference exposure range for adult patients was also simulated by random resampling from the actual subjects in the FCN-159-001 and FCN-159-002 studies.

Due to the constraints from the strength of the tablets (i.e., the lowest strength is 1 mg tablet), doses can only be administrated as an integer (e.g., 2, 3, 4, … mg). Therefore, dosing bands were explored with the aim of balancing between 1) exposure variability within each band, and 2) minimizing the deviation from the theoretical optimal dose (e.g., to match adult exposure; if the actual optimal dose for a hypothetical pediatric patient was 2.4 mg, the patient however could only be given a 2 mg dose which results in a 17% deviation from the actual optimal dose). Each dosing band had defined lower and upper boundaries for BSA, and all patients falling within that BSA range would get the same fixed dose. As expected, within each dosing band, the patients who are at the BSA boundary will have the largest deviation from their actual optimal doses. Therefore, a series of initial dosing bands are set as 0.5–1.5 mg, 1.5–2.5 mg, 2.5–3.5 mg and so on and the corresponding BSA boundaries were calculated accordingly using population mean exposure in adults and fixed effect’s PK parameters. Exposure for pediatric subjects (*n* = 500 in each band) and adult subjects (*n* = 500) were then simulated based on fixed integer dose levels (i.e., 2, 3, 4 mg and so on). PK exposure metrics of steady-state areas under the curves (AUC_24h, ss_) and maximum concentrations (C_max, ss_) were computed from the simulated PK profiles. The PK exposures (AUC_24h, ss_) in children within each band were compared with the simulated adult reference exposures (AUC_24h, ss_) using box plots. The BSA boundaries were further adjusted manually, and the final dosing bands were fine-tuned iteratively to achieve optimal matching to the adult exposure by visual inspection.

## 3 Results

### 3.1 Data summary

The final PopPK dataset consisted of 45 subjects, including 33 and 12 subjects from the FCN-159-001 and FCN-159-002 studies, respectively. The median age of the analysis population was 49 years (range, 20–71 years; Supplementary Table S2). A total of 1090 PK samples were available, with 60 (5.5%) below the limit of quantification. Given the low number of samples below the limit of quantification, they were excluded from the final analysis.

### 3.2 PopPK modeling

**FIGURE 1:**
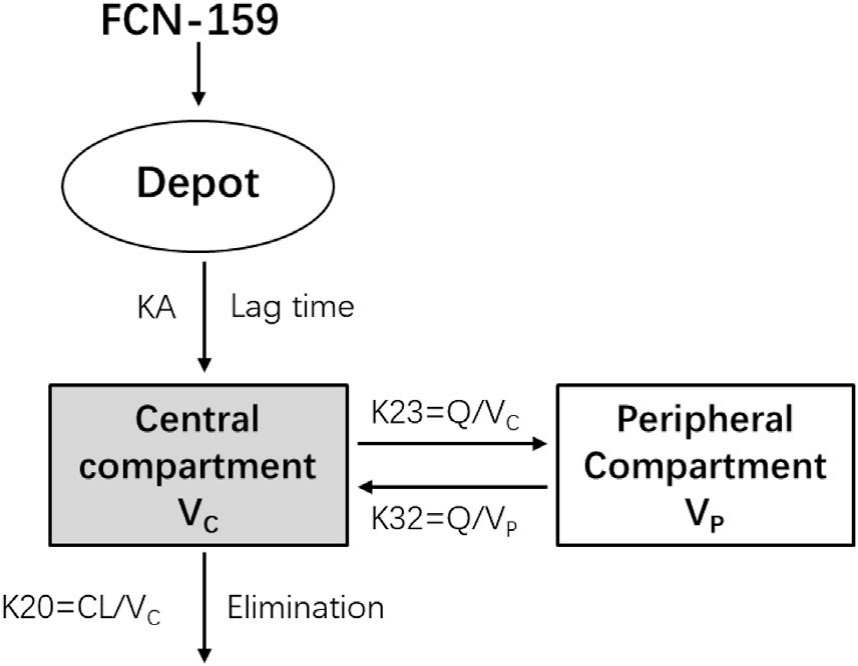
The base structure of the population pharmacokinetic model.

A two-compartment model with first-order absorption with lag time and linear elimination was selected as the best base structural model. The base model provided a good description of the PK data of FCN-159. The residual error was modeled as a proportional residual variability. This model was implemented using the PREDPP subroutine ADVAN4 TRANS4 and successfully converged with FOCE-I (Figure 1).

### 3.3 Covariate model and final model development

Besides BSA, other statistically significant covariates included in the model were: age, albumin on CL/F, total protein on V_C_/F, sex on V_P_/F, and cancer type on F1. The parameter estimates for age and albumin on CL/F were very small, i.e., −0.00972 (RSE: 23.70%) and −0.0134 (RSE: 54%), respectively. Therefore, those two covariates on clearance were removed from the final model. The removal of these two covariates resulted in slight increases of IIV in CL/F (from 17.2% to 19%) and OFV (from 2995.8 to 3014.724). The final covariate model included the effects of BSA on CL/F, V_C_/F, Q/F and V_P_/F; of total protein on V_C_/F; of sex on V_P_/F; and cancer type on F1. The final PK parameter and covariate relationships are presented in Table 1.

**TABLE 1:** The final PK parameter and covariate relationships.

PK parameter	Covariate relationship
${{}_{CL}}_{/F_{i}}$	$\theta {}_{1}\times e^{\eta 1}\times {\left({BSA}_{i}/1.66\right)}^{{{}_{\theta }}_{8}}$
${{{}_{V}}_{{}_{C}/F}}_{i}$	$\theta {}_{2}\times e^{\eta 2}\times {\left({BSA}_{i}/1.66\right)}_{}^{{{}_{\theta }}_{11}}\times {\left({TP}_{i/68.95}\right)}^{{{}_{\theta }}_{12}}$
${{}_{Q/F}}_{i}$	$\theta {}_{3}\times e^{\eta 3}\times {\left({BSA}_{i}/1.66\right)}^{{{}_{\theta }}_{9}}$
${{}_{V}}_{{{}_{P}}_{i}}$	$\theta {}_{4}\times e^{\eta 4}\times {\left({BSA}_{i}/1.66\right)}_{}^{{{}_{\theta }}_{10}}\times \left(1+\theta {}_{13}\times SEX{}_{i}\left(if\;female\right.\right))$
${{}_{F}}_{{{}_{1}}_{i}}$	$1\times \left(\theta {}_{7}\times TYPE{}_{i}\left(if\;NF1\right.\right))$

*CL/Fi, V*_*C*_ */Fi, Q/Fi,* and *V_P_/Fi* were PK, parameters for subject ith; sex (SEXi) was categorized into male (SEXi = 0) and female (*SEXi* = 1); cancer type (*TYPEi*) was categorized into melanoma (*TYPEi* = 0) and NF1 (*TYPEi* = 1).

**FIGURE 2:**
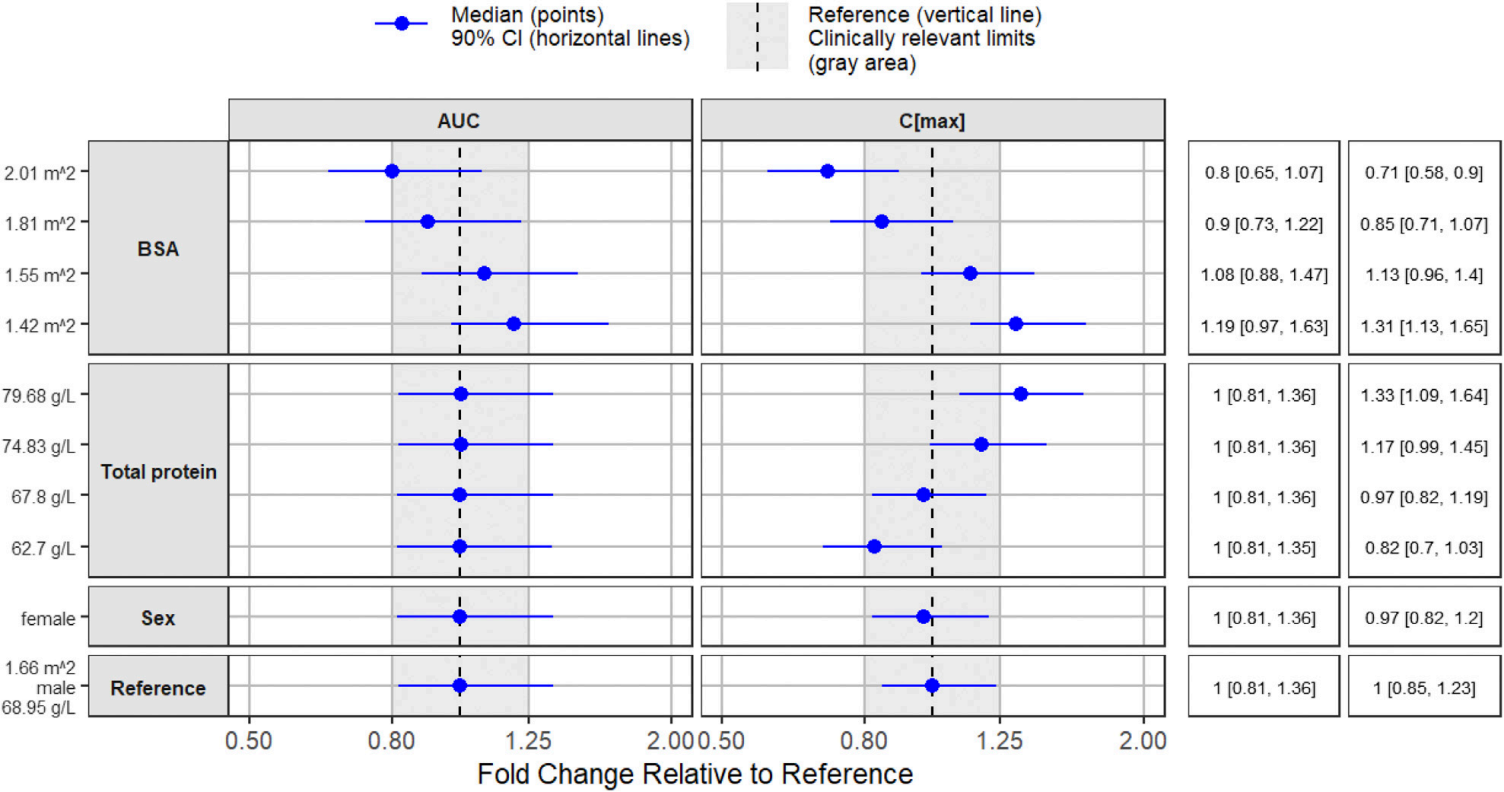
Effects of covariates on FCN-159 exposures (steady state AUC and C_max_).

Higher BSA was associated with lower FCN-159 exposure. Typical subjects with BSA values of 1.42 and 2.01 m^2^ (corresponding to 5th and 95th percentiles of the population) would be expected to have 19% higher and 20% lower AUC_0-24, ss_ and 31% higher and 29% lower C_max, ss_, respectively, relative to a typical subject with a BSA of 1.66 m^2^. The impacts of total protein and sex on AUC_0-24, ss_ were minimal. However, a higher total protein would lead to a higher C_max_. For instance, a patient with total plasma protein of 76.89 g/L would be expected to have 30% higher C_max, ss_ relative to a patient with total plasma protein of 68.95 g/L. Male patients would have approximately 3% higher C_max, ss_ relative to female counterparts. The effects of each covariate on FCN-159 exposures (AUC_0-24, ss_ and C_max, ss_) are illustrated in forest plots (Figure 2).

### 3.4 Model evaluation

The condition number of the final model was 101.64, indicating the model was stable. The estimated PopPK parameters from the bootstrap (1000 bootstrap runs) are presented in Table 2, and the 95% CI was computed based on all converged bootstrap runs. The final model’s parameters were estimated with reasonable precision (RSE% generally less than 20%) except for estimates for covariate effects. The parameter estimates of the final model all fell within the 95% CI of the bootstrap. Therefore, the model was considered robust.

**TABLE 2:** Final population pharmacokinetic model parameters for FCN-159.

	Final model parameter estimates		Bootstrap estimates	
Fixed effect parameter				
	Estimate	RSE	Mean	95% CI
CL/F (L/hr)	13.2	3.80%	13.2	12.2–14.2
V_C_/F (L)	48.7	13.30%	48	35.6–61.8
Q/F (L/hr)	35.1	7.70%	35.2	29.8–40.5
V_P_/F (L)	314	6.10%	317	273–355
KA (1/hr)	0.5	8%	0.494	0.421–0.58
ALAG1 (hr)	0.211	2.10%	0.216	0.192–0.23
F1*	1.29	6.50%	1.3	1.11–1.47
CL/F - BSA	1.11	26.70%	1.08	0.495–1.72
Q/F - BSA	2.20	22.10%	2.19	1.2–3.2
V_P_/F - BSA	3.84	13.30%	3.8	2.8–4.89
V_C_/F - BSA	2.41	43.60%	2.3	0.0948–4.72
V_C_/F - TP	−3.1	30.60%	−3.06	-5–1.2
V_P_/F - SEX	0.751	24.80%	0.75	0.386–1.12
Between subject variability				
IIV CL/F	19%	13.5%	18.3%	13.4%–23.3%
IIV V_C_/F	69.4%	15.2%	67.2%	46.1%–86.6%
IIV Q/F	26%	18.3%	24.9%	15.0%–33.5%
IIV V_P_/F	26.5%	15.5%	25.0%	17.1%–33.3%
Residual variability				
Proportional residual	25.8%	5.5%	25.7%	22.8%–28.5%

RSE, Relative standard error; Mean, Arithmetic mean; CI, Confidence interval; CL/F, Clearance; V_C_/F, Central volume of distribution; V_P_/F, Peripheral compartment volume of distribution; Q/F, Apparent distribution clearance. * F1 is the relative bioavailability for NF1, where F1 is 1 for melanoma.

**FIGURE 3:**
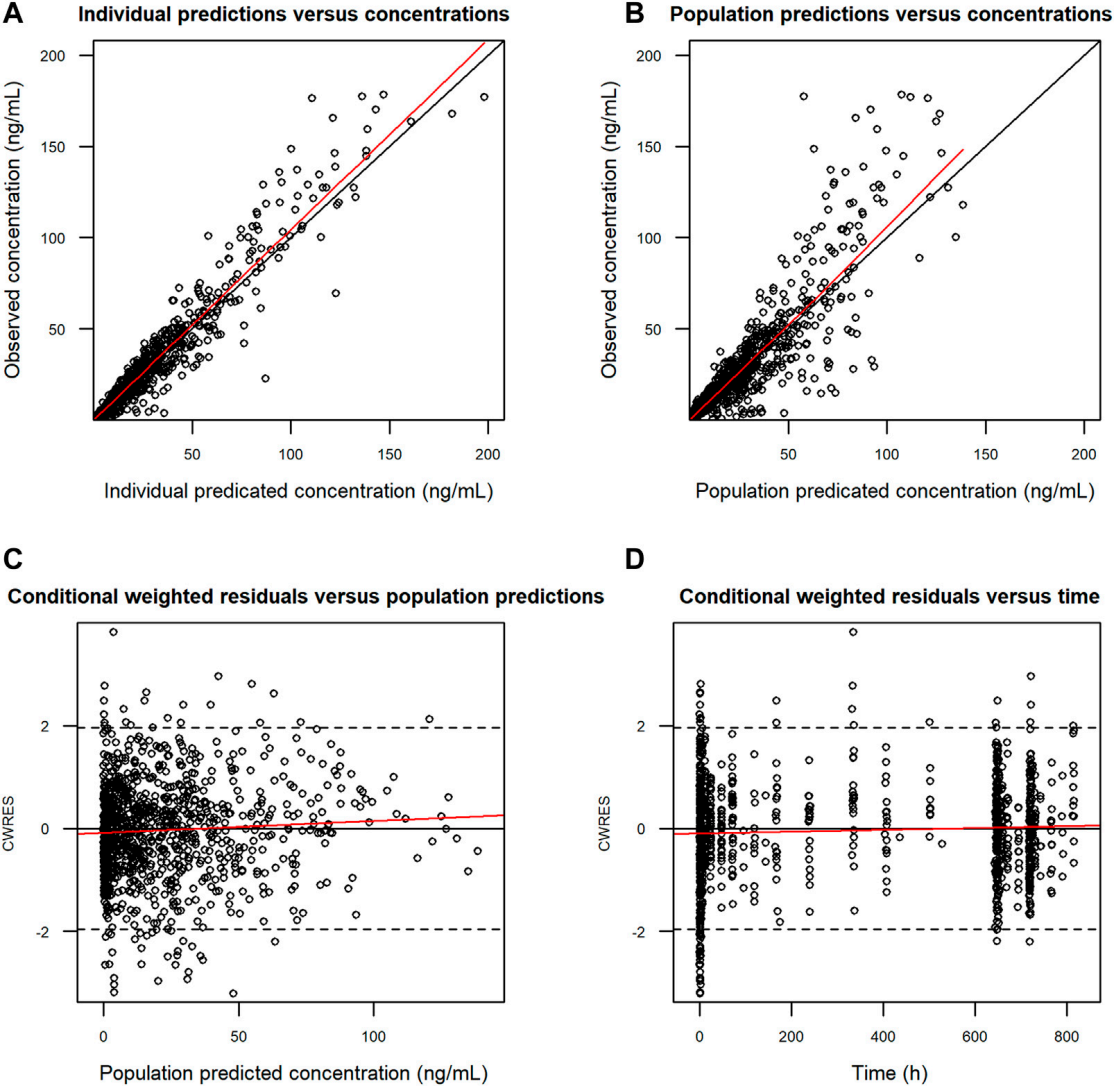
Goodness-of-fit of the final PK model for FCN-159. **(A)** Observed (DV) *vs*. individual predicted concentrations (IPRED). **(B)** Observed (DV) *vs*. population predicted concentrations (PRED). **(C)** Conditional weighted residuals (CWRES) *vs*. population predicted concentrations (PRED). **(D)** Conditional weighted residuals (CWRES) *vs*. time after doses (TAD). Black circles represent observations. The red lines show locally weighted scatterplot smoothing. The black lines represent the identity lines. PK, pharmacokinetics. Dots are individual data points, and the solid red lines are smoothed locally weighted smoothing (LOESS) lines. In the two plots in the left column row, solid black lines were lines of identity, while in the two plots in the right column, solid horizontal lines show the zero value.

**FIGURE 4:**
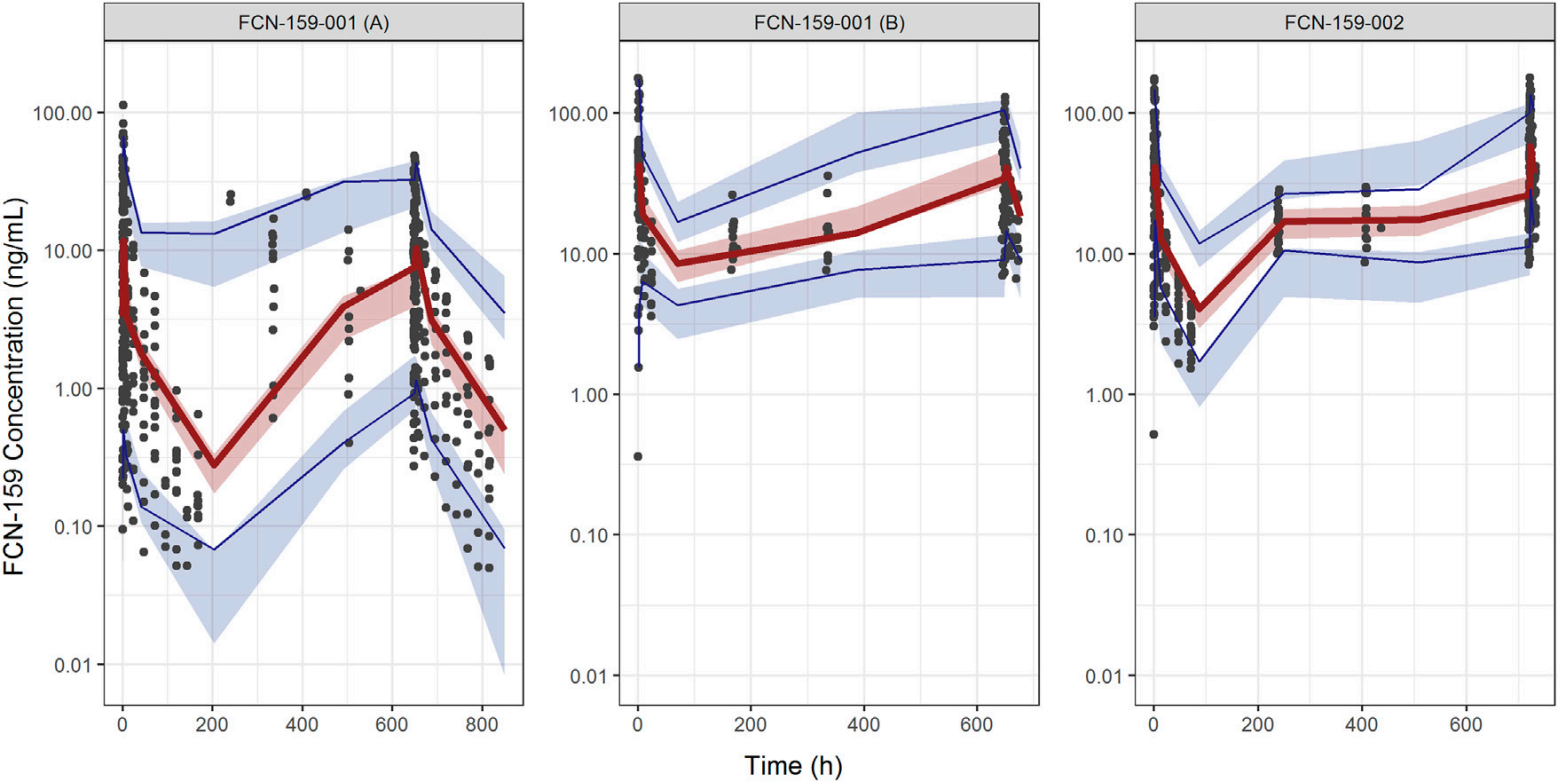
Visual predictive check plots for FCN-159. The Left and middle panels show the plots of the FCN-159-001 study, and the right panel shows the VPC plot of the FCN-159-002 study. The plots for FCN-159-001 are divided into two parts due to different PK sampling schedules and dose levels. Black dots are observed data. The red lines and the red shaded areas represent the median of observed data and the simulation-based 95% confidence intervals for the median of the predicted data, respectively. And the lower and upper blue lines and the blue shaded areas represent the 5th and 95th percentiles of observed data and the simulation-based 95% confidence intervals for the 5th and 95th percentiles of the predicted data, respectively.

Goodness-of-fit plots demonstrated a close agreement between the observed and predicted FCN-159 concentrations. (Figures 3A, B). Besides, conditional weighted residuals *vs*. population predicted values and times from the first dose were randomly distributed around the line of identity and showed no tendency supporting the appropriateness of the structural and residual error models (Figures 3C, D). The VPC results suggested that the final model was appropriate and sufficient to reproduce the time courses of FCN-159 plasma concentrations and their respective IIV in the patient populations (Figure 4). VPC stratification by study and population indicated the absence of readily apparent sources of heterogeneity in FCN-159’s PK by these factors.

### 3.5 Model simulation of BSA-based dosing strategy

**FIGURE 5:**
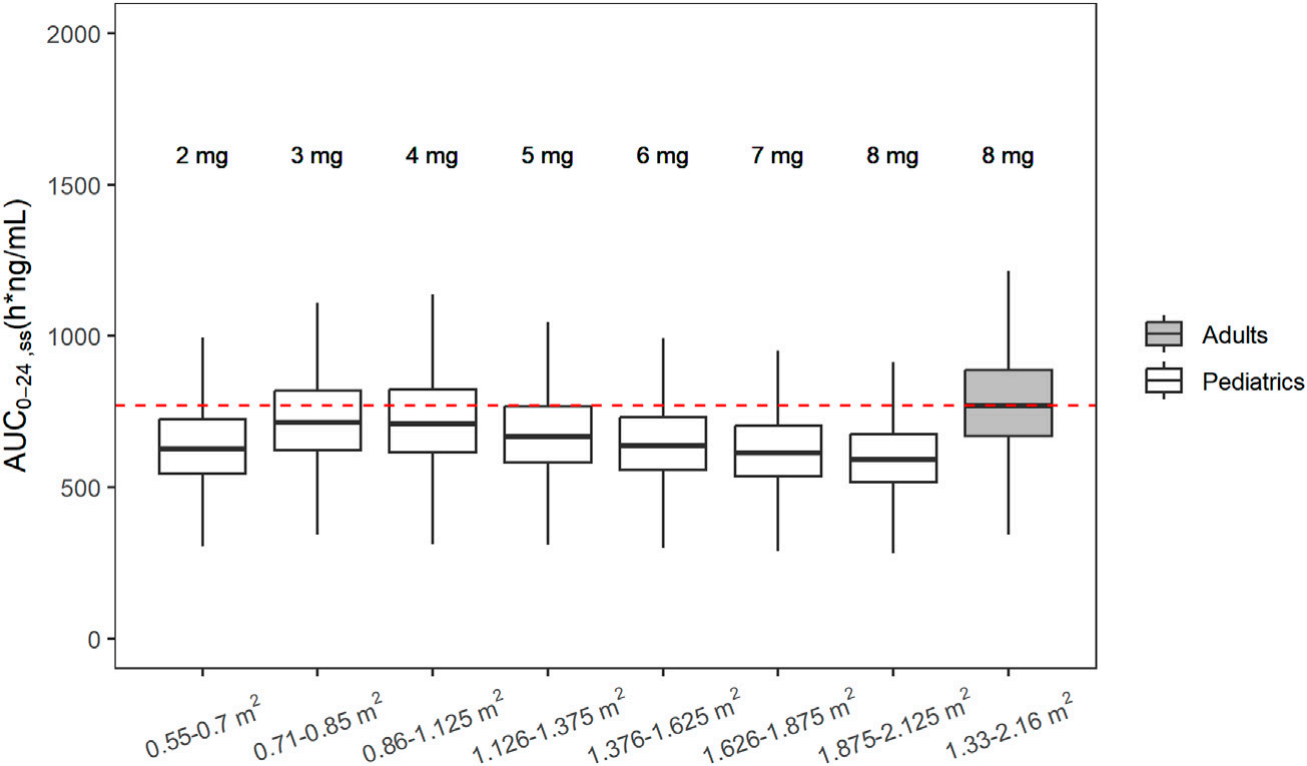
Simulated steady state AUC_0-24h_ following recommended doses in pediatric patients and adult with NF1. From left to right, white boxes correspond to 2, 3, 4, 5, 6, 7, and 8 mg fixed doses for pediatrics and the last gray box shows the simulated AUC_0-24h, ss_ from adult patients with NF1 after administration of RP2D (8 mg) in adults.

The developed population PK model for adults was adapted using allometric scaling to inform the pediatric dose selection *via* model simulation. Different BSA-based dose levels were simulated, and 4 mg/m^2^ was selected as a reasonable starting dose for pediatric patients, which provided slightly lower exposure compared with exposures observed in adults at RP2D 8 mg (Figure 5). Each dosing bin was created empirically by trial and error to convert the doses in each dosing bin to a flat fixed dose (i.e., in mg integer) due to the constraints from tablet strength. When matching the pediatric exposure to adults’, the key exposure metric was AUC_24h, ss_, with C_max, ss_ as the secondary metric. Simulated AUC_24h, ss_ values were summarized per BSA bracket and overlaid with the simulated AUC_24h, ss_ from the adult FCN-159-002 study. Overall, the distributions of AUC_24h, ss_ across the different dosing bands were comparable and overlapping.

## 4 Discussion

The PK of FCN-159 was adequately described by a 2-compartment model with linear first-order elimination from the central compartment and first-order delayed absorption. FCN-159 has low solubility and permeability and has been tentatively considered a Biopharmaceutical Classification System (BCS) class IV compound (data on file, Fosun Pharma). The T_max_ was quite short, ranging from 1 to 3 h. The absorption phase of the FCN-159’s PK is relatively erratic. Different absorption models (including delayed absorption, first-order, sequential zero- and first-order, transit compartment) were evaluated. Delayed absorption significantly improved the model’s fit. Compared with simple first-order absorption, none of the models with higher complexity provided significant improvement in terms of capturing the absorption profiles. Therefore, first-order absorption with first-order delay was chosen to describe the absorption phase of the FCN-159’s PK. Although the FCN-159-001 (melanoma patients) and FCN-159-002 (NF1 patients) studies used the same formulation, and higher exposure (both C_max_ and AUC) was observed in the NF1 population. As C_max_ is mainly driven by the volume of distribution whereas AUC is mainly impacted by clearance; when both parameters are impacted toward the same direction, a rational explanation is that bioavailability differed between two studies. Therefore, cancer type as a covariate was added to the bioavailability term in the model. The results indicated NF1 patients had roughly 30% higher bioavailability compared with melanoma patients, but the underlining mechanism remains unclear. One possibility for the lower absorption in melanoma patients may be the compromised gastrointestinal (GI) tract functions which is widely recognized for metastatic melanoma (Schuchter et al., 2000; Kohoutova et al., 2021). In contrast, NF1 is a relative benign tumor type that may have minimal impact on the GI functions. To fully understand the mechanism that drives the bioavailability differences between two indications, physiologically based pharmacokinetic modelling (PBPK) (Templeton et al., 2018) may be considered to test some potential mechanistic drivers such as differences in GI motility, etc. However, as NF1 is a rare disease which is difficult to enroll large number of patients for mechanistic investigation, more mechanistic modeling was not attempted. Nevertheless, lower bioavailability in melanoma patients is also consistent with tolerability data showing that melanoma patients have higher MTD (12 mg) compared with NF1 patients (8 mg). The FCN-159-002 study had daily continuous dosing, and PK profiles from the study did not show the two-compartment PK behavior due to the long terminal half-life of FCN-159 (31.8–57.4 h). Inclusion in the FCN-159-001 study that had the single dose PK run-in design was critical to fully characterize FCN-159’s PK. Overall, the parameters of the final PK model were precisely estimated.

An allometric scaling approach was used to project pediatric PK using the established adult PK model. BSA and BW were tested as the allometric terms and resulted in quite similar goodness of fit. BSA was selected in this study as an allometric term, as it is more widely used for starting dose selection in the medical oncology community (Moreno et al., 2017), which was also used in the posology of the approved MEK inhibitor selumetinib (Schalkwijk et al., 2021). Allometric scaling terms with exponents of 0.75 and 1 were recommended to account for the effects of body weight on clearance and volume of distribution, respectively (Anderson and Holford, 2008; Holford et al., 2013). In contrast, there is no well-defined recommendation for exponent values for BSA. Majority of equations for BSA calculation involve both weight and height. Hence, height in the equation prevents the direct conversion from the exponent values of weight to those of BSA. Livingston, et al. (Verbraecken et al., 2006) defined a relationship between BSA and weight as BSA (m^2^) = 0.1173 x BW (kg)^0.6466^. In this equation, BSA is only a function of body weight. Using this equation, exponent values of 0.75 and 1 on weight would translate to exponent values of 1.16 and 1.54 on BSA. The estimated exponents from the modeling dataset were 1.11 and 3.84 for apparent clearance and volume, respectively. The large difference between the estimated and empirically exponents for volume may be due to the limited BSA ranges included the modeling dataset. The current dataset contained BSA values ranging from 1.33 to 2.16 m^2^. The target population for the prediction had an estimated BSA range from 0.55 to 2.125 m^2^, which is much wider than the current modeling dataset. Nevertheless, a larger exponent, if overestimated, would result in an underestimation of volume of distribution prediction in young pediatric patients. As C_max_ is affected by the volume of distribution and is mainly related to safety, a potential overestimation of C_max_ would give a relatively conservative starting dose selection. In contrast, the estimated exponent for clearance is more consistent with the empirical value. Therefore, AUC prediction is likely to be more accurate than C_max_. When selecting the exposure metrics for matching, AUC was also chosen as the primary metrics because of the mode of the action of the drug. For FCN-159 to be effective, it requires constant inhibition of the target. The treatment for NF1 could last for years. For targeted antitumor therapy with chronic dosing, AUC is deemed more relevant than C_max_ from efficacy perspective. For safety, the main adverse events (AEs) observed for FCN-159 including folliculitis and paronychia (Hu et al., 2022) typically developed after several weeks of treatment indicating those AEs are not acute and likely to be more related to AUCs as well. Furthermore, comprehensive analysis including population PK, exposure-response, pediatric posology simulations were conducted for selumetinib, the first approved MEK inhibitor for NF1 treatment. The analysis showed that AUC was the most relevant exposure metrics for all endpoints (efficacy and safety) and therefore was chosen as the matching metrics for the proposed pediatric posology (Schalkwijk et al., 2021). Currently, a pediatric study in NF1 is ongoing with 4 mg/m^2^ as the starting dose recommended by the model simulation aiming to match the AUC_SS_ observed in adults at RP2D.

One limitation for the current modeling and extrapolation approach is the allometric scaling was only applied to clearance and volume of distribution. For drug absorption, model extrapolation assumes the bioavailability stays the same across the entire age of interest. For pediatrics aged 2 years and older, the key GI physiological characteristics that impact drug absorptions are quite similar to adults, such as gastric pH (Stewart and Hampton, 1987), gastric emptying (Morselli et al., 1980; Butler et al., 1994), and intestinal transit (Strolin Benedetti and Baltes, 2003). Nevertheless, compared to this population PK model, a PBPK model capable of incorporating age dependent GI physiological terms can probably provide more mechanistic extrapolation on FCN-159 absorption in pediatrics. To mitigate the potential risk due to uncertainty in bioavailability hence leading to over or underpredicted exposure in pediatrics, in the FCN-159 pediatric trials, patients are enrolled in a stepwise manner from higher age to lower age. The real time PK data will inform if the model or dose adjustment is warranted to account for age dependent effect on bioavailability.

FCN-159 metabolism is mainly mediated by three CYP enzymes, including CYP3A4, CYP2C19 and CYP2C8. The expression and activities of these three CYP enzymes are expected to approach adult levels by 2 years of age. Thus, the organ maturation function was not included in the pediatric model (Liu et al., 2017). In addition to body size, the covariate model identified sex and total plasma protein as significant covariates. Total plasma protein has a relatively large effect on the volume of distribution. FCN-159 has high plasma protein binding affinity (99.6%) (data on file, Fosun Pharma). For high-affinity protein binding drugs, the unbound fraction could differ at different drug concentrations. Due to the challenge of measuring low free drug concentration in the protein binding assay, the concentration-dependent effect on plasma protein binding cannot be accurately assessed experimentally. However, as the covariate model indicated, higher plasma protein concentration is expected to lead to higher FCN-159 binding in plasma and less penetration in peripheral tissues, which results in smaller volume of distribution. The impact of plasma total protein on C_max_ is moderate (20%–30%) and unlikely to be clinically meaningful. So, no dose adjustment based on total protein is deemed necessary.

Simulations were performed in the pediatric population with the goal of obtaining FCN-159 plasma exposure similar to that observed at the RP2D in NF1 adults (Abduljalil et al., 2014). For dose selection, the same pharmacokinetics/pharmacodynamics relationship between children and adults was assumed based on the previous understanding of another MEK inhibitor, selumetinib, approved for NF1 treatment. Selumetinib did not show age-dependent exposure-response (i.e., exposure-efficacy and exposure-safety) in patients between 3 and 18 years old (Schalkwijk et al., 2021). The results of these simulations using the allometry adapted model successfully showed comparable exposures between pediatric patients and adults within each dosing band, which provided the basis for posology selection for initial pediatric trials.

## 5 Conclusion

Overall, the analyses described herein represent a comprehensive effort to use modeling and simulation approaches to extrapolate PK data of a MEK1/2 inhibitor from adults to pediatric patients for pediatric dose selection. FCN-159’s adult PK was described by a 2-compartment model with linear first-order elimination and first-order delayed absorption. Aside from the body size effect (BSA) on apparent clearance and volume and the patient’s effect on bioavailability, other covariates of interest, including sex, age, plasma protein and renal/hepatic function markers did not have clinically meaningful effects on FCN-159’s PK. Simulations from the final model were used to inform dose selection for pediatric dose development. BSA-based dosing bands were recommended, which is predicted to result in exposure that is comparable to the adult exposure at the clinically recommended dose.

## Data Availability

The original contributions presented in the study are included in the article/Supplementary Material, further inquiries can be directed to the corresponding author.
