# Supplementary material for: Population pharmacokinetics of FCN-159, a MEK1/2 inhibitor, in adult patients with advanced melanoma and neurofibromatosis type 1 (NF1) and model informed dosing recommendations for NF1 pediatrics
Source: Front Pharmacol. 2023 Jan 23;14:1101991. doi: 10.3389/fphar.2023.1101991 (PMC9899833; doi:10.3389/fphar.2023.1101991)
Supplement: Supplementary file 1 [file DataSheet1.docx]

**Supplementary Table 1.** Summary of Studies Included in the Population PK Analysis of FCN-159

| Study number, Clinical Trials. gov identifier | Study Design | Dose, formulation, and regimen | PK sampling |
| --- | --- | --- | --- |
| FCN-159-001, [NCT03932253](https://clinicaltrials.gov/show/NCT03932253) | A multi-center, open-label, single-arm phase Ia dose exploration and phase Ib dose expansion study to evaluate the safety, tolerability, pharmacokinetics, and preliminary anti-tumor activity of FCN-159 in advanced melanoma patients with aberrant NRAS (phase Ia) and advanced melanoma patients with NRAS mutation (phase Ib) | Phase Ia dose-escalation study：  0.2, 0.5, 1,2, 4, 6, 8, 12 and 15 mg  Oral, once a day, continuous treatment for 21 days, 7 days of rest, 28 days in a cycle. | Phase Ia, single ascending dose period：  D1 pre- and post-dose 0.5, 1, 1.5, 2, 3, 6 and 12 h; D2 or post-dose 24 h; D3 or post-dose 48 h; D4 or post-dose 72 h.  Phase Ia, multiple dosing period：  C1D1 (Day 4 after first dose) pre-dose; C1D8 pre-dose; C1D15 pre-dose; C1D28 pre- and post-dose 0.5, 1, 1.5, 2, 3, 6 and 12 h; C2D1 pre-dose (C1D28 post-dose or 24 h).  Phase Ib dose expansion study:  C1D1 pre-dose; C2D1 pre-dose and post-dose 2 h; C2D2 (C2D1 post-dose 24 h) pre-dose; C3D1 pre-dose; C4D1 pre-dose. |
| FCN-159-002, [NCT04954001](https://clinicaltrials.gov/show/NCT04954001) | A multi-center, open-label, single-arm phase I dose exploration and phase II dose expansion study to evaluate the safety, tolerability, pharmacokinetics, and anti-tumor activity of FCN-159 in adult and pediatric patients with neurofibromatosis type 1. | Phase I study：  Cohort 1: Adult patients:  Dose escalation period：  4 mg, 6 mg, 8 mg, and 12 mg | Phase I study：  Cohort 1: Adult patients：  Cycle1, C1: C1D1 pre- and post-dose 0.5, 1, 2, 3, 4, 6 and 10 h；C1D2 pre-dose, C1D8 pre-dose, C1D15 pre-dose, C1D28 pre- and post-dose 0.5, 1, 2, 3, 4, 6 and 10 h. Cycle2, C2: C2D1: pre-dose (C1D28 post-dose 24 h). |

**Supplementary Table 2.** Demographics and Baseline Characteristics for the Population PK Analysis dataset

| Characteristics | FCN-159-001 (N=33) | FCN-159-002 (N=12) | Total (N=45) |
| --- | --- | --- | --- |
| Age, years, median (range) | 55 (34-71) | 26.5 (23-57) | 49 (20-71) |
| Male, n (%) | 16 (48.48) | 9 (75) | 26 (54.17) |
| Body weight, kg, median (range) | 67 (46-106) | 56.5 (43-88) | 63 (43-106) |
| Diagnosis, n (%) |  |  |  |
| Melanoma | 33 (100) | - | 33 (68.75) |
| NF1 | - | 12 (100) | 12 (31.25) |
| Height, cm, median (range) | 162 (147-176) | 165 (146-177) | 162 (146-177) |
| BSA, m^2^, median (range) | 1.74 (1.36 - 2.16) | 1.64 (1.33-2.05) | 1.66 (1.33-2.16) |
| ALB, g/L, median (range) | 45.6 (35.2-50.8) | 45.0 (38.7-53) | 45.0 (35.2-53) |
| WBC, 10^9^/L, median (range) | 5.09 (2.33-12.8) | 6.56 (6.05-13.6) | 5.84 (2.33-13.6) |
| RBC, 10^12^/L, median (range) | 4.41 (3.25-5.31) | 4.99 (4.25-6.09) | 4.56 (3.25-6.09) |
| PLT, 10^9^/L, median (range) | 202 (130-381) | 226 (172-391) | 222 (130-391) |
| TP, g/L, median (range) | 72.5 (59-82) | 69.5 (61.3-80) | 71.75 (59-82) |
| ALT, U/L, median (range) | 16.3 (5-35.4) | 13 (4-32) | 14.8 (4-35.4) |
| AST, U/L, median (range) | 21 (10 - 49.7) | 17.5 (8-29) | 19 (8-49.7) |
| LDH, U/L, median (range) | 221 (122-1802) | 149 (112-247) | 209 (112-1802) |
| TBIL, μmol/L, median (range) | 11.3 (6-29.8) | 12.7 (4.6-22.8) | 12.2 (4.6-29.8) |
| CrCL, mL/min, median (range) | 104 (49.1-224) | 116 (73.5-174) | 104 (49.1-224) |
| eGFR, mL/min/1.73 m^2^, median (range) | 86.0 (51.1-209) | 108 (69.2-169) | 91.7 (51.1-209) |


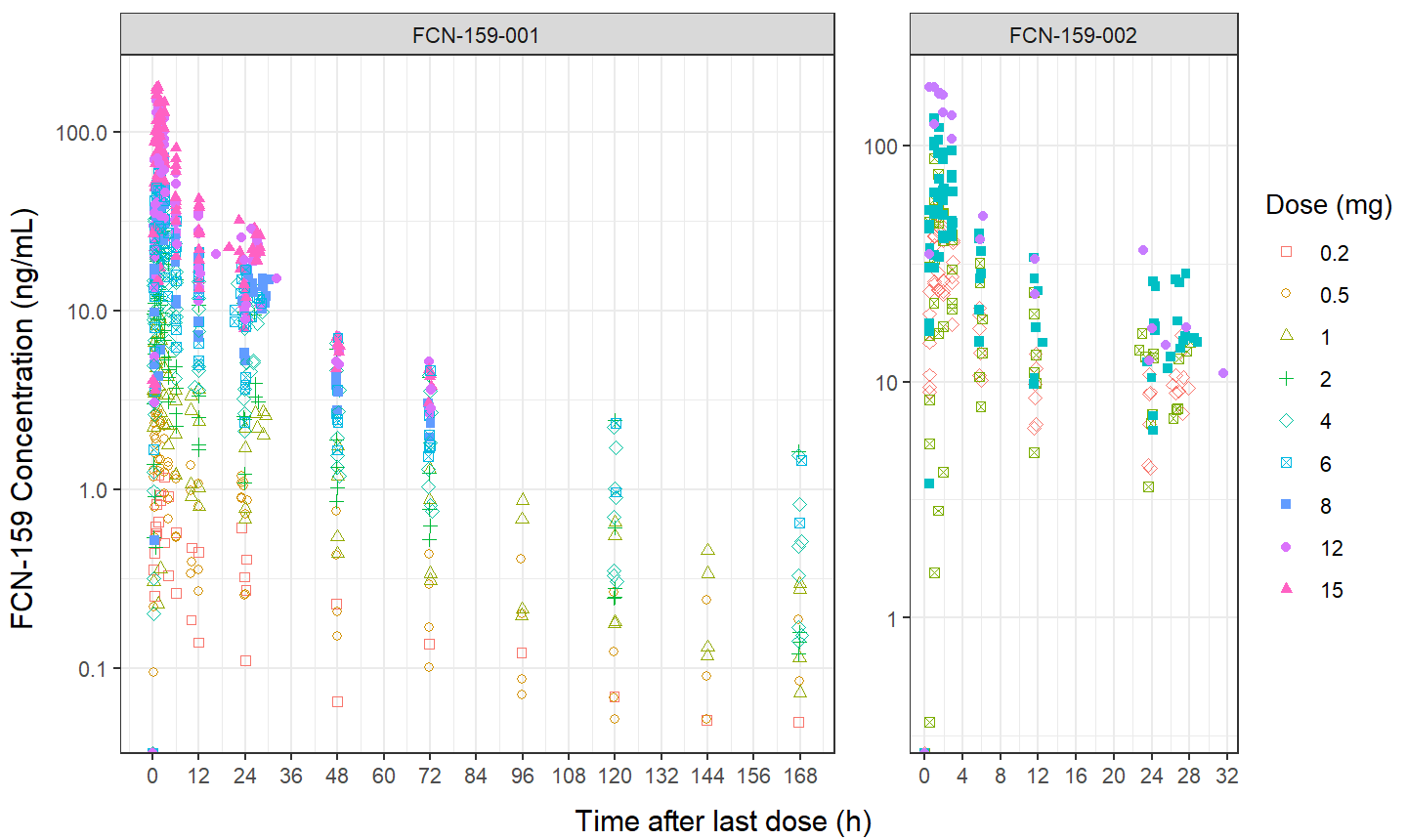


**Supplementary Figure 1.** Scatter plots of plasma concentration versus time after last dose of FCN-159 by dose group. The left and right panels show the plasma concentration – time profiles of FCN-159-001 and FCN-159-002, respectively. Each point represents the plasma drug concentration.
